# A guide for using NIH Image J for single slice cross-sectional area and composition analysis of the thigh from computed tomography

**DOI:** 10.1371/journal.pone.0211629

**Published:** 2019-02-07

**Authors:** Douglas E. Long, Alejandro G. Villasante Tezanos, James N. Wise, Philip A. Kern, Marcas M. Bamman, Charlotte A. Peterson, Richard A. Dennis

**Affiliations:** 1 College of Health Sciences and Center for Muscle Biology, University of Kentucky, Lexington, KY, United States of America; 2 College of Arts and Sciences, Department of Statistics, University of Kentucky, Lexington, KY, United States of America; 3 College of Medicine, Department of Radiology, University of Arkansas for Medical Sciences, Little Rock, AR, United States of America; 4 Department of Internal Medicine, Division of Endocrinology, and Barnstable Brown Diabetes and Obesity Center, University of Kentucky, Lexington, KY, United States of America; 5 Center for Exercise Medicine and Department of Cell, Developmental, and Integrative Biology, University of Alabama at Birmingham, Birmingham, AL, United States of America; 6 Geriatric Research, Education and Clinical Center, Central Arkansas Veterans Healthcare System, North Little Rock, AR, United States of America; 7 Donald W Reynolds Department of Geriatrics, University of Arkansas for Medical Sciences, Little Rock, AR, United States of America; University of Washington, UNITED STATES

## Abstract

Reports using computed tomography (CT) to estimate thigh skeletal muscle cross-sectional area and mean muscle attenuation are often difficult to evaluate due to inconsistent methods of quantification and/or poorly described analysis methods. This CT tutorial provides step-by-step instructions in using free, NIH Image J software to quantify both muscle size and composition in the mid-thigh, which was validated against a robust commercially available software, SliceOmatic. CT scans of the mid-thigh were analyzed from 101 healthy individuals aged 65 and older. Mean cross-sectional area and mean attenuation values are presented across seven defined Hounsfield unit (HU) ranges along with the percent contribution of each region to the total mid-thigh area. Inter-software correlation coefficients ranged from R^2^ = 0.92–0.99 for all specific area comparisons measured using the Image J method compared to SliceOmatic. We recommend reporting individual HU ranges for all areas measured. Although HU range 0–100 includes the majority of skeletal muscle area, HU range -29 to 150 appears to be the most inclusive for quantifying total thigh muscle. Reporting all HU ranges is necessary to determine the relative contribution of each, as they may be differentially affected by age, obesity, disease, and exercise. This standardized operating procedure will facilitate consistency among investigators reporting computed tomography characteristics of the thigh on single slice images.

**Trial Registration:** ClinicalTrials.gov NCT02308228.

## Background

Skeletal muscle accounts for approximately 40–45% of body mass and plays a vital role in health and disease through its influences on energy metabolism, glucose utilization, and physical function. Skeletal muscle is a remarkably plastic tissue continuously adapting to physiological and pathological conditions. The most potent modulators of the skeletal muscle phenotype are contraction and load, experienced during endurance and resistance exercise, whereas skeletal muscle loss is exacerbated by injury, illness, disuse, insufficient nutrient intake, and aging. This dramatic ability of skeletal muscle to adapt to various stimuli and its connection to morbidity and mortality make it the center of investigation in frailty, cachexia, sarcopenia, and metabolism-related research [1,2]. Thus, accurate measurements of muscle mass, size, and composition are critically important.

Methods for assessing skeletal muscle in vivo have evolved such that many previously complex techniques can now be utilized to provide precise comprehensive information into this tissue [3]. Of these, computed tomography (CT) is a well-accepted research method for assessing muscle size and volume (cross-sectional area (CSA) from 1 slice vs. multiple slices, respectively), as well as muscle composition (quality). A review and basic background for the use of CT in the assessment of human body composition has been previously described [4]. Briefly, the imaging modality provides quick high-resolution images differentiating soft tissues based on x-ray attenuation values called Hounsfield units (HU). Further attributes of CT have allowed researchers the ability to determine intermuscular fat infiltration and determine skeletal muscle lipid content based on the mean attenuation through the tissue [5]. Thus, muscle with lower attenuation values or low density (LDM) has a higher lipid concentration than does normal density muscle (NDM) and cut-points have been used by investigators to distinguish the two [6,7]. CT objectively monitors tissue composition with high sensitivity making it beneficial to aid in assessing small changes that may occur from resistance training, nutritional interventions, or disease. Analysis of CT images using software packages, however, can be quite time consuming and requires technical knowledge and analytical expertise that may not be readily available to researchers. Therefore, developing an accurate standardized approach for analysis of muscle quality and area by single slice CT will facilitate progress and enable comparison of data among investigations and across studies.

Inconsistencies currently exist in the literature for HU attenuation cutoffs to segment fat from muscle, and a HU attenuation range to define low density muscle area is often not included. Fat tissue is demarcated as an attenuation range of -190 HU to -30 HU, but a consensus has not been reached for skeletal muscle attenuation, with researchers defining it within an attenuation range as broadly as -29 HU to 150 HU or as narrowly as 0 HU to 100 HU, or even 35 HU to 100 HU. In older individuals, -29 HU to 199 HU might also be appropriate [8]. A review by Aubrey et al. discusses this methodological flaw and concludes that differential HU range utilization in image analysis may have a significant impact on the calculation of muscle CSA [9]. Further, a detailed method for data processing for research has not been widely accepted. To our knowledge, the first guide for clinicians to measure abdominal circumference and muscle using Image J was only recently published [10].

The purpose of this report is to provide a new, detailed description with easy to follow instructions for the use of NIH Image J to quantify CT images of the thigh that is semi-automated. We validate the procedure against SliceOmatic software, specifically developed for easy analysis of body composition from medical images, using a large dataset derived from a healthy, predominately Caucasian, older population, so that the relative contribution of each HU range is calculated consistently [8].

## Methods

### Subjects

CT acquisition occurred as part of the MASTERS study (Clinical Trials # NCT02308228), described in detail elsewhere [11]. Briefly, written informed consent was obtained during a University of Kentucky IRB approved protocol where individuals aged 65 and older received two 5-mm thick, bilateral, single slice CT scans at the mid-thigh defined as the midpoint between the inguinal crease and the proximal border of the patella. One hundred and one baseline scans were analyzed in this report.

### CT acquisition

CT images were obtained without contrast using a soft tissue algorithm, 120 kVp and 100 mA, 512 x 512 matrix, and a field of view (FOV) adjusted to capture the entire thigh of both legs. Each individual was supine with feet wrapped to avoid movement and wore non-constrictive clothing to reduce compression of soft tissue. In addition, legs were separated for ease of analysis. Each image was reviewed for quality so that movement, compression of the skin, and metal artifacts were not present. Once images were analyzed using Image J as described in detail below, a subset of 52 de-identified images were evaluated by the commercial software program, SliceOmatic, for comparison where the technical details of that analysis are described by Dennis et al. [8].

### Establishment of Hounsfield Unit (HU) ranges

**Fig 1:**
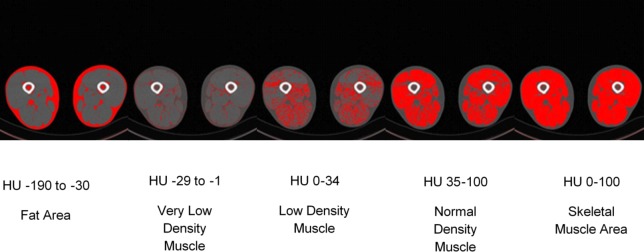
Graphical representation of various Hounsfield Unit ranges used for determining fat and skeletal muscle. Visual output from Image J when assessing fat area, very low density muscle (VLDM), low density muscle (LDM), normal density muscle (NDM), and skeletal muscle area (SKM1).

CT scanners are calibrated under the assumption that the HU attenuation value of 0 and -1000 are water and air, respectively. For the purposes of this tutorial, we are proposing that each HU attenuation range be reported separately so the total thigh CSA is accounted for. We subjectively define each HU attenuation range as the following: normal density fat (NDF), -190 to -30; very low density muscle (VLDM), -29 to -1; low density muscle (LDM), 0–34; normal density muscle (NDM), 35–100; high density muscle (HDM), 101–150; very high density muscle (VHDM), 151–199; bone, ≥ 200; LDM+NDM, skeletal muscle 1 (SKM1), 0–100; and VLDM+LDM+NDM+HDM, skeletal muscle 2 (SKM2), -29-150. SKM1 and SKM2 represent widely divergent, but common ranges used to report muscle area. Fig 1 shows a graphical representation of some of these defined ranges.

### Step-by-step methods for quantification of cross-sectional area (CSA) of soft tissue compartments of the thigh using Image J software

Image J can readily be downloaded from the NIH website (https://imagej.nih.gov/ij/download.html) and support many file formats including DICOM files. Depending on how your facility saves its CT images, DICOM files may be compressed in which a few extra steps are needed within Image J. Performing initial calibration checks of the image by noting the field of view (FOV) units and proper scaling is essential to ensuring data quality and must be performed prior to assessing CSA of the region of interest. The thigh or marrow space region of interest (ROI) is specified by using the wand selection tool or the polygon selection tool (if thighs are touching) once a threshold has been set using the adjust threshold function. ROI total areas are measured using the “analyze” and “measure” functions which will report area, minimum, maximum, and mean HU (attenuation) by default. The “image” and “adjust threshold” function allows the user to specify specific limits for their HU ranges which will highlight in red different tissue depots depending on the limits set. S1 Table shows step-by-step instructions for downloading Image J and using the adjust threshold function within the software for the assessment of soft tissue depots from one slice at the mid-thigh. The bone marrow area is excluded from this analysis to increase precision for fat and skeletal muscle area measurements as marrow contains HU ranges similar to that of other tissue types. Areas are calculated within the software by multiplying the number of pixels counted within a specified HU range by the pixel area determined by the FOV settings (pixel count x pixel width^2^). S2 Table shows the macro script developed for the semi-automation of the steps described in the S1 Table. This approach is applied to S1 and S2 File so that the results generated by our group can be checked and replicated. S1 File includes a de-identified CT image while S2 File includes the excel data file generated from the analysis of the image.

### Statistical analysis

Statistics were performed on 101 subjects with baseline CT images to assess the mean, standard deviation, and range of mid-thigh CT characteristics as well as age, BMI, and percent body fat determined by dual energy x-ray absorptiometry (DXA). Linear regression was used to determine the strength of the association between tissue depot areas between two available softwares commonly used for CT image analysis; NIH Image J and SliceOmatic. Bland-Altman plot analysis was performed to test the level of agreement between the current tutorial analysis protocol utilizing Image J and validated against edited SliceOmatic data to account for overlapping densities between muscle and fat with skin and marrow [8] using SAS version 9.4.

## Results

### Subject characteristics, mean attenuation and cross-sectional area (CSA) of the mid-thigh

Table 1 shows reference data for CT characteristics, including CSA and mean attenuation, at the mid-thigh acquired using NIH Image J software and the method shown in S1 and S2 Tables. This cohort consisted of 57 females and 44 males ranging in age from 64–91 with an average age of 70.2. The cohort was primarily non-obese (BMI ≤ 30) with a mean BMI of 26.2, but had a broad range of total body fat percentage (18.7% - 50.9%) with females having significantly higher body fat percentages compared to males (39.3% vs. 30.4%). Mean total CSA of the mid-thigh was approximately 216 cm^2^ with approximately 90 cm^2^ and 111 cm^2^ attributed to fat (HU range -190 to -30) and skeletal muscle (SKM1, HU range 0–100), respectively, which accounts for 93% of the total area. Using the wider skeletal muscle attenuation range (SKM2, HU range -29 to +150), 119 cm^2^ is attributed to muscle accounting for 96.7% of the total area when combined with fat. Bone accounts for approximately 2.3% of the CSA. Mean muscle attenuation was 45.9 HU for SKM1 and 42.3 HU for SKM2. Our data confirm previous reports that a cut-point of 34 should be used to determine low vs. normal density muscle in older individuals. When the cohort was separated out by sex, significant differences were found for all CSA CT related variables excluding VLDM (HU range -29 to -1). Mean CT attenuation of VLDM, NDM, and SKM2 also differed by sex.

**Table 1:** Baseline demographics and computed tomography characteristics of study subjects.

Demographics	Females (n = 57) Mean ± SD (range)	Males (n = 44) Mean ± SD (range)	All (n = 101) Mean ± SD (range)	*p*
Age (years)	69.5 ± 3.8 (65.0–85.6)	71.3 ± 5.7 (64.4–91.2)	70.2 ± 4.7 (64.4–91.2)	0.07
BMI (kg/m^2^)	25.6 ± 3.4 (18.6–33.9)	27.0 ± 2.6 (18.7–41.6)	26.2 ± 3.1 (18.6–41.6)	0.03*
DXA Fat (%)	39.3 ± 6.3 (25.6–50.9)	30.4 ± 5.3 (18.7–33.9)	35.5 ± 7.4 (18.7–50.9)	< .0001*
**CT Mid-Thigh Cross-Sectional Areas (cm**^**2**^**)**				
Total Area (TA)	223.0 ± 42.1 (138.1–327.9)	206.9 ± 27.1 (142.7–260.4)	216.0 ± 37.1 (138.1–327.9)	0.02*
Normal Density Fat Area (NDF)	118.0 ± 37.8 (51.5–229.9)	53.9 ± 18.6 (23.1–113.1)	90.1 ± 44.4 (23.1–229.9)	< .0001*
Very Low Density Muscle (VLDM)	7.0 ± 2.6 (2.8–16.4)	7.8 ± 2.1 (4.2–15.4)	7.3 ± 2.4 (2.8–16.4)	0.07
Low Density Muscle Area (LDM)	22.3 ± 7.8 (10.2–50.1)	29.0 ± 8.3 (17.0–53.2)	25.2 ± 8.7 (10.2–53.2)	< .0001*
Normal Density Muscle Area (NDM)	69.4 ± 13.9 (40.5–111.2)	108.0 ± 19.1 (69.4–152.4)	86.2 ± 25.2 (40.5–152.4)	< .0001*
High Density Muscle Area (HDM)	0.3 ± 0.19 (0.07–0.92)	0.60 ± 0.24 (0.15–1.43)	0.40 ± 0.25 (0.07–1.43)	< .0001*
Very High Density Muscle Area (VHDM)	0.08 ± 0.03 (0.04–0.18)	0.12 ± 0.04 (0.05–0.23)	0.1 ± 0.04 (0.04–0.23)	< .0001*
Bone Area	4.6 ± 0.42 (3.7–5.6)	5.9 ± 0.60 (4.7–7.1)	5.1 ± 0.83 (3.7–7.1)	< .0001*
Skeletal Muscle Area (SKM1)#	91.7 ± 14.4 (61.0–140.0)	137.0 ± 19.6 (94.2–170.5)	111.4 ± 28.1 (61.0–170.5)	< .0001*
Skeletal Muscle Area (SKM2)^+^	99.0 ± 15.1 (69.4–147.9)	145.4 ± 20.4 (99.4–181.9)	119.2 ± 29.0 (69.4–181.9)	< .0001*
**Mean CT Attenuation (HU)**				
Total Area (TA)	-10.9 ± 18.3 (-54.6–31.9)	41.0 ± 14.7 (2.4–75.8)	11.7 ± 30.8 (-54.6–75.8)	< .0001*
Normal Density Fat (NDF)	-105.4 ± 3.9 (-118.4–98.1)	-95.6 ± 5.4 (-106.6–83.3)	-101.1 ± 6.7 (-118.4–83.3)	< .0001*
Very Low Density Muscle (VLDM)	-14.5 ± 0.60 (-15.6–13.2)	-14.1 ± 0.37 (-15.0–13.4)	-14.3 ± 0.54 (-15.6–13.2)	0.0006*
Low Density Muscle (LDM)	21.7 ± 0.79 (19.4–23.1)	22.2 ± 0.56 (20.5–23.3)	21.9 ± 0.73 (19.4–23.3)	0.001*
Normal Density Muscle (NDM)	52.9 ± 2.2 (48.1–58.6)	53.2 ± 1.9 (48.9–57.8)	53.0 ± 2.1 (48.1–58.6)	0.58
High Density Muscle (HDM)	117.6 ± 4.2 (109.9–128.3)	117.0 ± 3.1 (111.8–124.3)	117.3 ± 3.8 (109.9–128.3)	0.42
Very High Density Muscle (VHDM)	174.7 ± 3.2 (166.6–180.5)	174.0 ± 3.3 (165.6–181.2)	174.4 ± 3.2 (165.6–181.2)	0.36
Bone	1225.6 ± 110.6 (1044.4–1725.6)	1250.5 ± 82.3 (1101.0–1725.6)	1236.5 ± 99.6 (1044.4–1546.7)	0.20
Skeletal Muscle (SKM1)#	45.3 ± 3.7 (33.4–52.1)	46.6 ± 3.0 (38.6–52.8)	45.9 ± 3.5 (33.4–52.8)	0.08
Skeletal Muscle (SKM2)^+^	41.4 ± 4.5 (27.2–49.8)	43.6 ± 3.6 (33.2–50.6)	42.3 ± 4.3 (27.2–50.6)	0.01*

BMI, body mass index; DXA, Dual-energy x-ray absorptiometry; CT, computed tomography

# SKM1 represents the area between a HU range of 0–100 and is calculated as LDM + NDM.

+ SKM2 represents the area between a HU range of -29-150 and is calculated as VLDM + LDM + NDM + HDM.

* p < 0.05 between males and females.

### Percent contribution of each Hounsfield Unit (HU) range to the total mid-thigh CSA

Table 2 shows the relative contribution of individually defined soft tissue depots. NDF (HU range -190 to -30) contributes roughly 41% of the total mid-thigh area. SKM1 (HU range 0–100) encompasses the majority of the muscle area of the thigh, with NDM (HU range 35–100) accounting for 41% and LDM (HU range 0–34) contributing 12%. VLDM (HU range -29 to -1) contributes 3.5% of the total area, with a range of 1.5–7.3%. The HU range of 101–150 (HDM) contributes 0.2% on average to the total area, with the contribution of the range 151–199 (VHDM) being negligible.

**Table 2:** Percent contribution of defined HU ranges to the overall total area of the mid-thigh.

Defined Depot	Hounsfield Unit Range	Percent Contribution	Range
Normal Density Fat (NDF)	-190 to -30	40.53%	12.59–71.00%
Very Low Density Muscle (VLDM)	-29 to -1	3.41%	1.47–7.30%
Low Density Muscle (LDM)	0–34	11.75%	4.80–25.23%
Normal Density Muscle (NDM)	35–100	40.92%	13.32–67.85%
High Density Muscle (HDM)	101–150	0.20%	0.04–0.88%
Very High Density Muscle (VHDM)	151–199	0.05%	0.02–0.16%
Bone	≥200	2.44%	1.35–4.46%

HU, Hounsfield Unit

### NIH Image J vs. SliceOmatic

**Fig 2:**
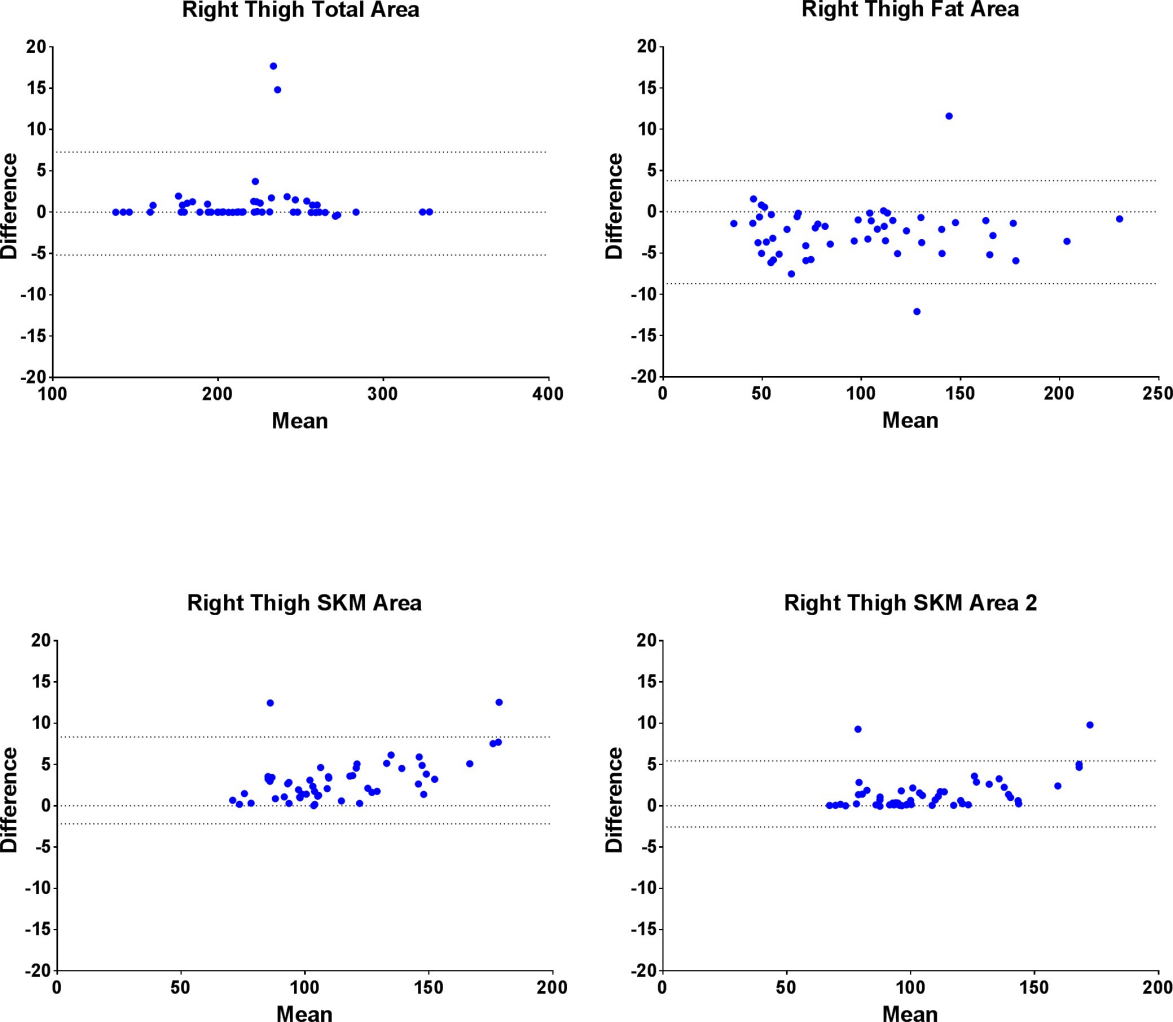
Bland-Altman plots of the individual subject differences between softwares for various cross sectional areas.

Table 3 shows the mean ± standard deviation (SD) values for the CSA measured from NIH Image J and SliceOmatic imaging analysis softwares. Intra-software correlation coefficients of R^2^ = 0.99 were seen between all specific areas measured except VLDM where the correlation coefficient was R^2^ = 0.92 due to SliceOmatic software editing. Bland-Altman plots in Fig 2 display the individual subject mean CSA differences between the two softwares with 95% confidence intervals for mid-thigh total area, fat area, SKM1 and SKM2 areas.

**Table 3:** Mean (SD) CT cross-sectional area measurements between NIH Image J and Slice-O-Matic software using current methods.

CT Mid-Thigh Cross-Sectional Area Measurement (cm^2^)	NIH Image J	Slice-O-Matic	R^2^
Total Area (TA)	219.93 (40.93)	219.52 (40.97)	.99
Normal Density Fat Area (NDF)	97.81 (45.96)	100.65 (45.93)	.99
Very Low Density Muscle (VLDM)	6.95 (2.33)	5.30 (1.97)	.92
Low Density Muscle Area (LDM)	24.26 (8.58)	23.35 (8.28)	.99
Normal Density Muscle Area (NDM)	83.84 (24.16)	83.49 (24.21)	.99
Skeletal Muscle Area (SKM1)	108.10 (26.90)	106.84 (26.27)	.99
High Density Muscle Area (HDM)	0.39 (0.22)	0.39 (0.22)	.99
Skeletal Muscle Area (SKM2)	115.44 (27.99)	112.53 (26.87)	.99
Very High Density Muscle Area (VHDM)	0.10 (0.04)	0.10 (0.04)	.99

Bone was not included due to technical differences in analysis. Image J data excludes bone marrow while SliceOmatic data includes marrow as bone area and edits overlapping densities between skin, muscle, and fat.

## Discussion

CT quantifies a variety of unique body composition characteristics in physiology research. However, the analysis of the produced images using complex or costly software systems may hinder the use of this technique. Two software packages are used regularly in the literature for the assessment of body composition by CT; SliceOmatic and NIH Image J, each having their own advantages and disadvantages that have been fully described elsewhere [12]. SliceOmatic comes with a very user-friendly interface and includes technical support, but contains costly start-up and yearly licensing fees. In addition, SliceOmatic allows the user to define HU ranges to delineate tissues such as fat, muscle, and bone. On the other hand, NIH Image J is freely available, but is limited to online developer resources making it difficult for a new user to effectively use the software. The NIH Image J website offers an online instruction manual for the various functions found within the software but none to our knowledge related to muscle size and composition determination. Typically, investigators using Image J must segment the region of interest by carefully tracing along the perimeter. Raw data (pixel counts) from the 16-bit user-traced region of interest is then exported into an excel program with automated area calculations for the determination of each tissue type based on pre-defined HU ranges using the histogram function (7–9). However, details of this process have not been fully described nor standardized. Here, we provide a step-by-step process for analyzing single-slice CT images of the mid-thigh using NIH Image J for the measurement of muscle and fat areas as well as mean attenuation in each soft tissue depot. This technique was chosen for three reasons: 1) to essentially eliminate inter-observer error; 2) to ensure correct and proper image calibrations within Image J rather than calculating it in an excel file; and 3) to threshold or segment HU ranges in an automated fashion within the program similar to SliceOmatic. Previously reported inter-observer coefficients of reliability between softwares are very high with values normally ranging from 0.98 to 1.00 for all comparisons between CSA measurements [12,13]. By utilizing the wand tool (as long as there is separation between the thighs), manual tracing differences between investigators are eliminated. Furthermore, Image J should automatically calibrate images to the proper scale based on the pixel width and pixel area; this eliminates the need to change pixel width in pre-set excel files as this could drastically affect calculated CSA data if it is not corrected for the field of view (FOV) of the obtained image. While we specifically measure the total mid-thigh region, this tutorial can be applied to individual muscle groups of the thigh, such as the vastus lateralis. More research is required to determine if this protocol will accurately quantify other anatomical regions of interest.

Large standardized reference data sets for both CSA and attenuation values of various anatomical regions across different populations according to age, sex, and race are needed. We provide standard reference CSA and attenuation values of a large dataset of relatively healthy older adults at the mid-thigh. Our data seem to be in line with those that have previously reported total thigh areas and total thigh muscle densities in a large older population cohort [14]. Additionally, we present the data in such a way that provides context to methodological considerations and discrepancies found among different investigations in hopes that a standardized procedure and consensus can be developed. Aubrey et al. highlights that while adipose tissue depots are consistently defined as a HU range of -190 to -30, definitions of skeletal muscle area range from a lower boundary of -29 HU to an upward boundary of 150 HU and occasionally even up to 200 HU [8,9]. Others have defined skeletal muscle within a smaller range of 0–100 HU [6,15–17]. This method ultimately leaves the HU range of -29 to -1 undefined and unmeasured which, in the abdomen, may account for as much as 13.5% of the total area in some individuals [9]. In our sample, we found that -29 to -1 HU range accounted for roughly 3.5% of the mid-thigh and may be as high as 7% in an older population. This is consistent with our recent results showing this HU range accounted for approximately 4% of the thigh area [8]. Dennis et al. also reported that this area increased with resistance training, supporting the conclusion that it should be included in muscle CSA measurements. However, this HU range seems to be in locations of transition such as around the edges of the skin and the muscle fascia calling into question the usefulness of including this transitional range. The HU ranges of 101–150 (HDM, 0.2%) and 151–199 (VHDM, 0.05%) contributed very little in this population to the total area measured by ImageJ which remains consistent with Dennis et al. using SliceOmatic. However, HDM is significantly impacted by resistance training providing a rationale for including this as muscle area [8]. Higher percentages may be seen in younger, more athletic populations and needs further investigation.

Mean attenuation of the muscle from CT has become a vital indicator of health related outcomes and prognosis. Goodpaster et al. were the first to show that skeletal muscle attenuation relates to lipid stores within the muscle leading to disruptions in metabolism such as insulin resistance [5,7]. Since then, several factors, such as age, obesity, diabetes, and surgical procedures are associated with reductions in muscle attenuation by 3–15 HU, but can be improved through strength and endurance training [9]. More recently, skeletal muscle attenuation has been examined in clinical populations, related to cancer progression and survival [18,19]. The utility of muscle attenuation measured by CT to provide insight into physical function, metabolism, and disease requires further study.

## Limitations

A limitation of this study is that CT scans were obtained from an older population that was generally healthy, predominately white, and had mostly normal or overweight BMIs. Generalizations to younger adults, or older adults with health conditions associated with obesity should be taken with caution as higher fat depots introduce measurement error. However, significant bias was not seen with abdominal visceral fat measures in abdominally obese individuals using either software package [12]. In addition, a general weakness of performing a single slice CT scan is that a detailed and precise standard operating procedure must be in place to position the scanner in order to get reliable, longitudinal values. To increase reliability, a scout scan should be performed so that anatomical landmarks of the femur can be used to determine the thigh midpoint.

## Conclusion

The tissue density range used to define muscle and fat varies between studies and areas of intermediate density are often omitted. This CT tutorial provides valuable step-by-step instructions for using free, but technically challenging, software to quantify both muscle size and composition in the mid-thigh, which was validated against a robust commercially available software. Image J and SliceOmatic generate similar results for the measurement of muscle and fat of all densities at the mid-thigh. We show that by evaluating individual HU sub-ranges, as much as 7.5% of overall total thigh area could be unaccounted for by omitting areas of intermediate density such as VLDM and VHDM. Thus, we recommend all individual HU ranges be evaluated as interests dictate to be the most inclusive of skeletal muscle and to determine responses due to aging and diseases processes, nutritional intervention, or exercise training. This standardized operating procedure will facilitate consistency among investigators reporting computed tomography characteristics of the thigh on single slice images.

## Supporting information

S1 Table Step-by-step methods for utilizing Image J to assess cross-sectional area and mean attenuation of the thigh. (DOCX)

S2 Table Generation of a macro to run semi-automated analysis of body composition. (DOCX)

S1 File CT Image. (DCM)

S2 File CT analysis of S1 file. (XLSX)

S3 File NIH Image J dataset. (CSV)

## Abbreviations

CT: computed tomography
CSA: cross-sectional area
HU: Hounsfield Unit
NIH: National Institutes of Health
kVp: peak kilovoltage
mA: milliamp
NDF: normal density fat
VLDM: very low density muscle
LDM: low density muscle
NDM: normal density muscle
HDM: high density muscle
VHDM: very high density muscle
DICOM: Digital Imaging and Communications in Medicine
FOV: field of view
ROI: region of interest
DXA: dual energy x-ray absorptiometry
