# Supplementary material for: A guide for using NIH Image J for single slice cross-sectional area and composition analysis of the thigh from computed tomography
Source: PLoS One. 2019 Feb 7;14(2):e0211629. doi: 10.1371/journal.pone.0211629 (PMC6366874; doi:10.1371/journal.pone.0211629)
Supplement: S1 Table — (DOCX) [file pone.0211629.s001.docx]

| **Download**  Download Image J from the NIH Image J website using the following link. It can be downloaded for Windows or Mac bundled with or without Java. https://imagej.nih.gov/ij/download.html |
| --- |
| **Open Image J**  Double click on the Microscope Icon. |
| **Opening uncompressed DICOM Images (other file formats are supported as well)**  1. Click File, Select Open.  2. Find uncompressed DICOM file of the image you would like to analyze saved on your computer.  3. Select Open.  **Image J cannot open a compressed DICOM file unless you download the plugin called Bio-Formats.  1. Download Plugin from the Image J plugin page.  2. Install Plugin.  a. Click Plugins in Image J Toolbar.  b. Select Install.  c. Find Bio-Formats in Image J folder on your computer, select open.  3. Opening a compressed DICOM file.  a. Click Plugins in Image J Toolbar.  b. Select Bio-Formats.  c. Click Bio-Formats Importer.  d. Find compressed DICOM file of the image you would like to analyze.  e. Select Open.  f. Click OK with Default Settings.  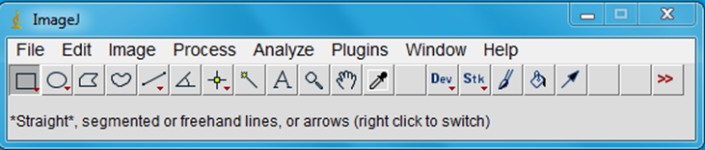 |
| **Initial Calibration Checks to Ensure Data Quality**  1. Ensure that the image is 16-bit.  a. Click Image in Image J Toolbar.  b. Select Type, 16-bit should be checked. (16-bit should also be showing in the image header)  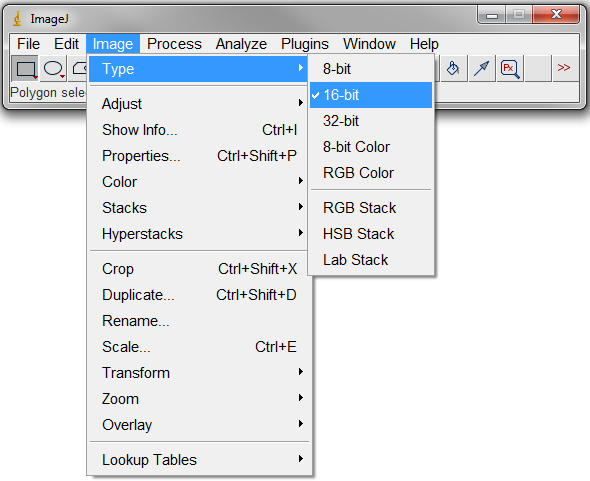 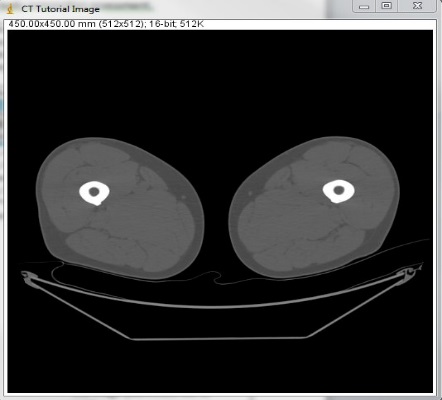  2. Ensure that the image is calibrated to the proper scale (should be automatically set). Notice image dimensions in the image header. To double check scale is correct:  a. Click Analyze in the Image J Toolbar.  b. Select Set Scale.  c. Enter 512 (in this case) for known distance in pixels.  d. Enter 450 (in this case) for known distance.  e. Change units to mm if necessary.  Scale = 512/450 = 1.1378 pixels/mm  Pixel width = 500/450 = 1.1111 mm/pixel  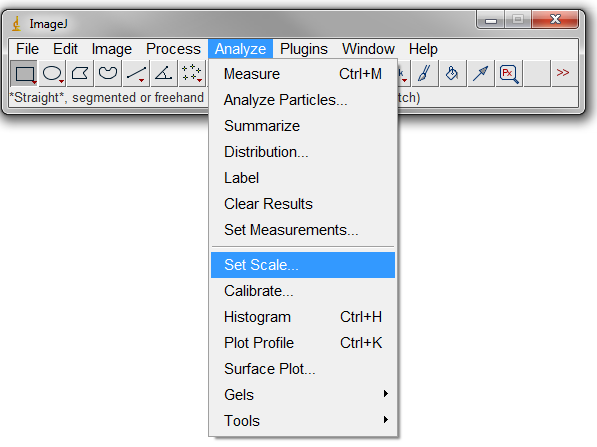 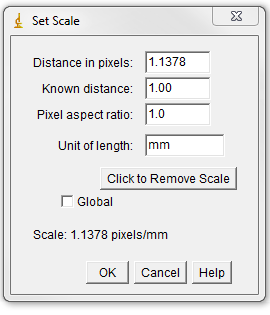 |
| **1. Assessment of Mid-Thigh Total Area (All HU included)**  1. Click Image in Image J Toolbar.  2. Select Adjust.  3. Click Threshold.  4. Click Set.  5. Set Lower Threshold to -190.  6. Set Upper Threshold to 2000 (this is arbitrary and does not matter much).  7. Click OK (this should highlight both thighs in red).  8. Select Wand Tracing Tool depicted by the arrow.  9. Click in Thigh you want to analyze (dominant or non-dominant)  a. This should trace the outline or border of the thigh.  10. Click Analyze in Image J Toolbar.  11. Select Measure.  a. This will display area of ROI, mean, min, and max thresholds (mm^2^).  ***Make sure the “limit to threshold” box is unchecked if previous images have been analyzed in the Analyze, Set Measurements Box, See next step***  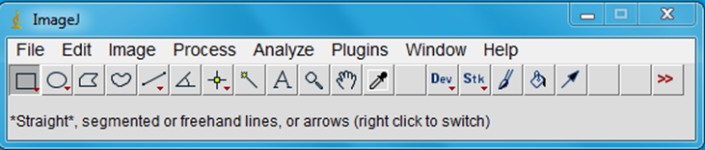 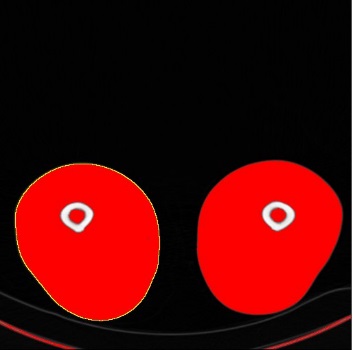 |
| **2-10. Thresholding for the Assessment of Soft Tissue Depots**  Very Low Density Muscle Area (VLDM) (Use Step 5A)  Low Density Muscle Area (LDM) (Use Step 5B)  Fat Area (Use Step 5)  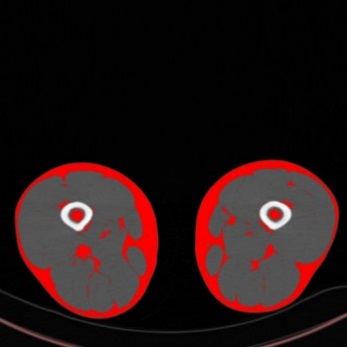 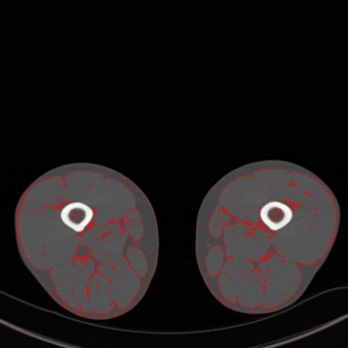 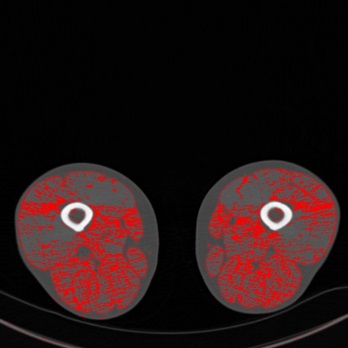  High Density Muscle Area (HDM) (Use Step 5E)  Total Skeletal Muscle Area (SKM1) (Use Step 5D)  Normal Density Muscle Area (NDM) (Use Step 5C)  **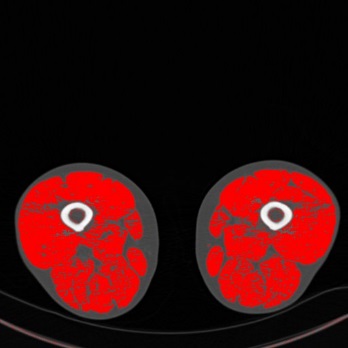 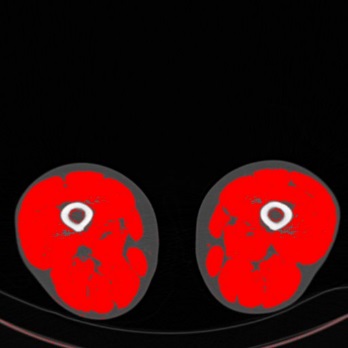 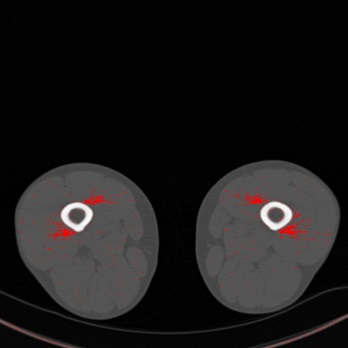**  Bone Area (Use Step 5H)  Very High Density Muscle Area (VHDM) (Use Step 5G)  Total Skeletal Muscle Area (SKM2) (Use Step 5F)    **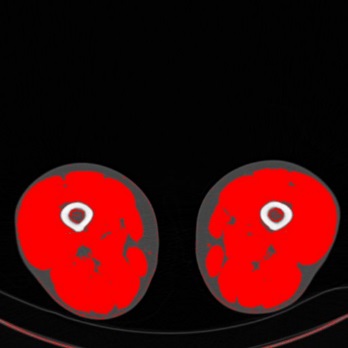 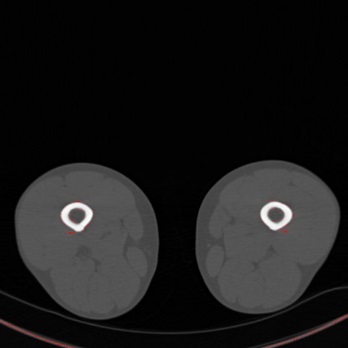 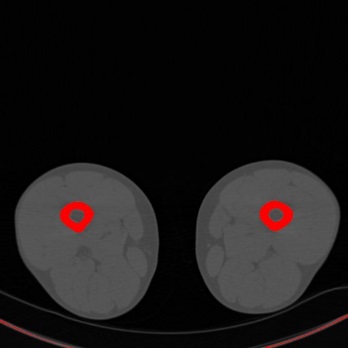**    1. Click Image in Image J Toolbar.  2. Select Adjust.  3. Click Threshold.  4. Click Set  5. Set Lower Threshold to -190. Set Upper Threshold to -30.  A. Set Lower Threshold to -29. Set Upper Threshold to -1.  B. Set Lower Threshold to 0. Set Upper Threshold to 30 (younger populations, ([Ross et al., 2002](#_ENREF_3))) or 34 (older populations, ([Chalé et al., 2013](#_ENREF_1); [Chomentowski et al., 2009](#_ENREF_2))).  C. Set Lower Threshold to 31 (younger populations) or 35 (older populations). Set Upper Threshold to 100.  D. Set Lower Threshold to 0. Set Upper Threshold to 100.  E. Set Lower Threshold to 101. Set Upper Threshold to 150.  F. Set Lower Threshold to -29. Set Upper Threshold to 150.  G. Set Lower Threshold to 151. Set Upper Threshold to 199.  H. Set Lower Threshold to 200. Set Upper Threshold to max value which can be found in row 1 of the results box. The max was 1656 in the results box shown below in save and export data. Setting at 2000 will usually be high enough.  6. Click OK.  7. Click Analyze in Image J Toolbar.  8. Select Set Measurements.  9. Check the Box “Limit to Threshold.”  10. Click OK.  *Steps 8-10 may be skipped if done previously. Remember to uncheck this box if calculating a total area within a defined region.  11. Click Analyze in Image J Toolbar.  12. Select Measure. This will display area of ROI, mean, min, and max thresholds in your defined threshold range (mm^2^). |
| **11. Assessment of Marrow Total Area**  1. Click Image in Image J Toolbar.  2. Select Adjust.  3. Click Threshold.  4. Click Set  5. Set Lower Threshold to -190.  6. Set Upper Threshold to 200 (this is arbitrary and does not matter much).  7. Click OK.  **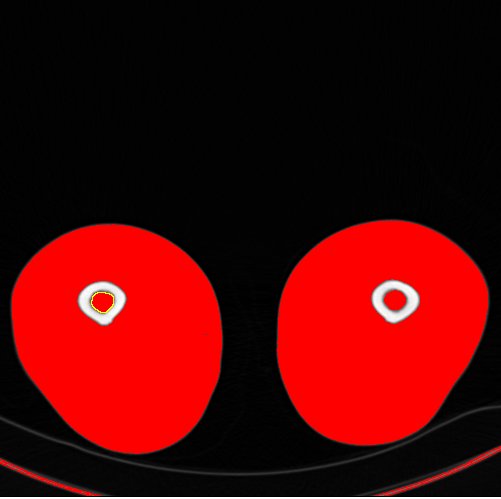**  8. Select Wand Tracing Tool.  9. Click in Marrow Area you want to analyze (dominant or non-dominant)  a. This should trace the area within the bone.  10. Click Analyze in Image J Toolbar.  11. Select Set Measurements.  12. Uncheck the Box “Limit to Threshold.”  13. Click OK.  14. Click Analyze in Image J Toolbar.  15. Select Measure.  This will display area of ROI, mean, min, and max thresholds (mm^2^)  11. Select Measure.  a. This will display area of ROI, mean, min, and max thresholds (mm^2^). |
| **12-19. Thresholding for the Assessment of Marrow Fat**  Marrow has similar pixel densities as both muscle and fat. In order to be more accurate, these pixels can be subtracted from each calculated range.  The same steps (5-5H) may be followed as the Mid-Thigh. Marrow Fat, HDF, LDM etc may then be subtracted from Mid-Thigh Fat for a more accurate assessment etc. |
| **Save and Export Data**  In Results Box:  1. Click Edit and Select All  2. Right Click on Highlighted Data  3. Copy  4. Paste in Excel CT Analysis Template  a. Paste in Field B6 or B35 for right or left leg.  b. Template automatically converts data to cm^2^ etc  5. Data in green are used for this analysis excluding the marrow.  **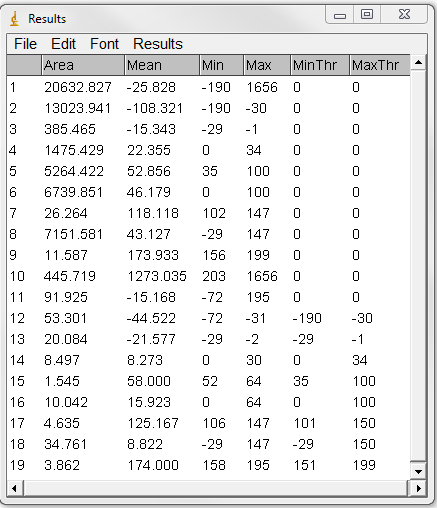** |

For the purposes of this tutorial, Image J Version 1.50i with default settings was used to open DICOM files. If mean attenuation is not a variable of interest, the assessment of SKM1 and SKM2 is not necessary as LDM and NDM can be combined in excel for SKM1, and VLDM, LDM, NDM, and HDM can be combined for SKM2. Intermuscular fat area can be obtained using the same steps described above for fat area by using the polygon tool to trace the outline of the muscle fascia (not shown). An excel template may be obtained by contacting the first author, Douglas Long.

_Chalé, A., Cloutier, G.J., Hau, C., Phillips, E.M., Dallal, G.E., Fielding, R.A., 2013. Efficacy of whey protein supplementation on resistance exercise-induced changes in lean mass, muscle strength, and physical function in mobility-limited older adults. The Journals Of Gerontology. Series A, Biological Sciences And Medical Sciences 68, 682-690._

_Chomentowski, P., Dubé, J.J., Amati, F., Stefanovic-Racic, M., Shanjian, Z., Toledo, F.G.S., Goodpaster, B.H., 2009. Moderate Exercise Attenuates the Loss of Skeletal Muscle Mass That Occurs With Intentional Caloric Restriction—Induced Weight Loss in Older, Overweight to Obese Adults. Journals of Gerontology Series A: Biological Sciences & Medical Sciences 64A, 575-580._

_Ross, R., Freeman, J., Hudson, R., Janssen, I., 2002. Abdominal obesity, muscle composition, and insulin resistance in premenopausal women. The Journal Of Clinical Endocrinology And Metabolism 87, 5044-5051._
