# Supplementary material for: A guide for using NIH Image J for single slice cross-sectional area and composition analysis of the thigh from computed tomography
Source: PLoS One. 2019 Feb 7;14(2):e0211629. doi: 10.1371/journal.pone.0211629 (PMC6366874; doi:10.1371/journal.pone.0211629)
Supplement: S2 Table — (DOCX) [file pone.0211629.s002.docx]

| **Generate New Macro**  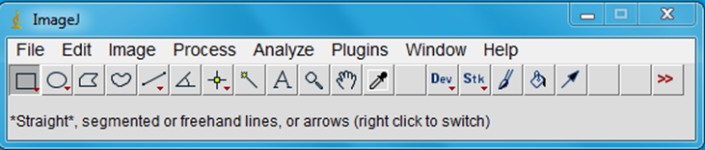 | 1. Click Plugins > New > Macro.  2. Copy and paste macros seen below into the text box. |
| --- | --- |
| **Macro for Thigh Assessments** | Copy and paste this Macro into the macro text box. This utilizes F1 to run the macro.  macro "Macro 1 [F1]" {  run("Set Measurements...", "area mean min redirect=None decimal=3");  setThreshold(-190, 200);  run("Measure");  run("Set Measurements...", "area mean min limit redirect=None decimal=3");  setThreshold(-190, -30);  run("Measure");  setThreshold(-29, -1);  run("Measure");  setThreshold(0, 34);  run("Measure");  setThreshold(35, 100);  run("Measure");  setThreshold(0, 100);  run("Measure");  setThreshold(101, 150);  run("Measure");  setThreshold(-29, 150);  run("Measure");  setThreshold(151, 199);  run("Measure");  setThreshold(200, 2500);  run("Measure");  setThreshold(-190, 200);  } |
| **Macro for Marrow Assessments** | Copy and paste this macro below macro 1. This utilizes F2 to run the macro.  macro "Macro 2 [F2]" {  run("Set Measurements...", "area mean min redirect=None decimal=3");  setThreshold(-190, 200);  run("Measure");  run("Set Measurements...", "area mean min limit redirect=None decimal=3");  setThreshold(-190, -30);  run("Measure");  setThreshold(-29, -1);  run("Measure");  setThreshold(0, 34);  run("Measure");  setThreshold(35, 100);  run("Measure");  setThreshold(0, 100);  run("Measure");  setThreshold(101, 150);  run("Measure");  setThreshold(-29, 150);  run("Measure");  setThreshold(151, 199);  run("Measure");  setThreshold(-190, 200);  } |
| **Save Macro** | 1. Click File, Save As.  2. Name File followed by .ijm to save the macro under the Image J folder in the macros or plugins folder. |
| **Install Macro** | 1. Click Plugins, Select Macro, Select Install.  2. Find Saved Macro, Click Open. |
| **Using Macro** | 1. Follow Figure 1 through initial checks and step 10 of the assessment of mid-thigh total area.  2. Push F1 on the keyboard to automate the generation of data relating to the thigh.  3. Push F2 (after tracing marrow area with wand tool) to automate and generate data relating to the marrow. |
